# Polymorphism of *MnSOD (Val16Ala)* gene in pregnancies with blighted ovum: A case-control study

**Published:** 2017-08

**Authors:** Asiyeh Moshtaghi, Hamidreza Vaziri, Reyhaneh Sariri, Hoorieh Shaigan

**Affiliations:** 1 *Department of Biology, Faculty of Sciences, University of Guilan, Rasht, Iran.*; 2 *Guilan University of Medical Sciences, Rasht, Iran.*

**Keywords:** Molecular genetic, Abnormalities, Blighted, Ovum, Abortion, Gestational sac, Superoxide dismutase

## Abstract

**Background:**

Blighted ovum is one of the most common reasons for abortion during the first three months of pregnancy. Manganese superoxide dismutase (MnSOD) is an important antioxidant enzyme in the human immune system. The gene is located on 6q25 chromosome and acts on mitochondrial matrix. In the case of mutation or inactivity of this enzyme, mitochondrial and nuclear DNA will severely be destructed. The most common polymorphism of its gene is Val16Ala.

**Objective:**

The aim was to investigate a possible mutation in pregnant women who had abortion during the first trimester of pregnancy due to blighted ovum.

**Materials and Methods:**

In this case-control study, 34 women were entered as the case and control groups, respectively. Genome DNA was extracted from saliva samples and its genotype was determined using Tetra-primer amplification refractory mutation system polymerase chain reaction technique.

**Results:**

In the case group, 16 (48%) cases had Val/Val genotype, 17 (50%) were heterozygote and had Val/Ala genotype, and 1 (2%) had Ala/Ala genotype. Among controls, 7 (22%) items had Val/Val genotype, 6 (17%) had Val/Ala genotype, and 21 (61%) had Ala/Ala genotype. The frequency of TT, CT, and CC genotypes was 48%, 50%, and 2% in case group and 22%, 17%, and 61% in control group, respectively. Statistical analysis revealed a significant relationship between Val16Ala polymorphism of MnSOD gene and blighted ovum (p= 0.0003).

**Conclusion:**

It has concluded that a significant relationship exists between Val16Ala polymorphism of MnSOD gene and blighted ovum.

## Introduction

Blighted ovum is one of the most common reasons for abortion during first three months of pregnancy (1). After fertilization of ovum with a sperm, the fertilized ovum will naturally plant in the womb and cell divisions commence (2, 3). Following placenta and the pregnancy sac formation, the embryonic division may stop causing anembryonic pregnancy or blighted ovum. Its reason is completely unknown but, it is suggested that genetic and chromosome disorder is the probable reason (4). An embryonic pregnancy is the main cause of about 50% of abortions during first three months of pregnancy. Although no embryo is formed, placenta continues to growth and pregnancy hormones will be secreted from placenta to mother’s blood (5). 

The disorder may be diagnosed through sonography at the end of the second month, which reveals the existence of an empty pregnancy sac with >20 mm diagonal. Alternations in a number of chromosomes may suspend cell division of primary zygote. It has been found that blighted ovum is the main reason of one-third of abortions before 8 wk of pregnancy (6-10). 

**Figure 1:**
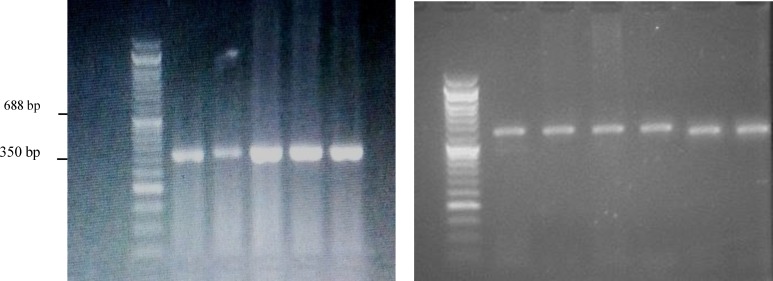
PCR products of T and C Alleles. A- PCR product of T-allele with 688 base pairs band. B- PCR product of C-allele with 350 base pairs band

It is known that mitochondrion is one of the most important places in the cell for aggregation of reactive oxygen species, and free radicals (11). Free radicals such as superoxide are among by-products of cellular respiration. The presence of MnSOD in mitochondrial matrix is a defensive mechanism of cells to remove radicals (11, 12). This is a homotetramer metalloprotein with four atoms of manganese in each subunit (Figure 1). The manganese atoms act as co-factors to facilitate the catalytic process (12). As most women tolerate severe stresses during pregnancy, providing stable conditions for them is highly required. It is necessary to follow the activity of antioxidants and genes producing them in their body. 

The aim of this study was to investigate polymorphism changes of MnSOD gene in women with a blighted ovum. The choice was based on its important role as one of the most efficient antioxidants in human cells and its significant activity changes during ovarian cycle and pregnancy.

## Materials and methods

This case-control study was performed on women referred to a Women Hospital, Guilan University of Medical Sciences and some private gynecologist offices during November 2015 to September 2016. 34 women with 20-38 years old who had to undergo abortion due to blighted ovum during the first trimester of pregnancy (gestational age of 8-13 wk) as the case group and 34 healthy women in the same age and gestational age range as the controls were enrolled in this study.

Our exclusion criteria was history of systemic diseases such as diabetes, kidney disorders, gastrointestinal diseases, and central nervous disorders. After a rinse with distilled water, about 5 ml of both group’s saliva was sampled in Falcon sterile tubes, immediately centrifuged and stored at -20^o^C for later experiments. 

In next stage, DNA was extracted by GPP solution (Sinaclon Company, Russia) and identified through spectroscopy and placed DNA on gel electrophoresis. DNA was then replicated through allele-specific polymerase chain reaction (PCR) using Tetra-primer amplification refractory mutation system PCR (ARMS-PCR) method and probable Val16Ala polymorphism in MnSOD and four replicated primer was investigated by Oligo 7 primer analysis software. Primers employed for the alleles were as follows: C: (5 CGGTAGCACCAGCACTAGCA3) Fc and (5 TGGAGCCCAGATACCCCAAAG3) RcT: (5 CCACTCAAGTACGGCAGAC3) Ft and (5 TGGAGCCCAGATACCCCAAAA3) Rt Taq enzyme, MgCl_2_ and dNTP buffer, water and DNA were employed to replicate required gene. PCR production for T-allele (the dominant allele) was a band with 688 base pairs and a band with 350 base pairs for C-allele (the recessive allele). 

Based on the results, all case and control samples were replicated and PCR process was done (once) for T-allele and C-allele. Each sample was analyzed on the basis of 350 and 688 base pairs bands. Samples with one 688 base pairs band are a healthy person with TT genotype. Those with both 350 and 688 base bp are CT heterozygote and the ones with one 350 bp have CC mutated homozygote (Figure 1).

**Ethical consideration**

The principle of the study was based on the Declaration of Helsinki. The Institutional Review Board of the University of Guilan approved the research protocol. The aim of the research was explained to the participants and informed consent was signed by each individual. 

**Statistical analysis**

Statistical analysis was performed using Madcalc (version 12/1) software. Each experiment was repeated 3 times and p<0.05 was assigned as significant.

## Results

The PCR products are presented in Figure 1. Among 34 samples with a blighted ovum, 7 (20%) cases had Val/Val genotype, 6 (17%) were heterozygote and had Val/Ala genotype and 21 (61%) had Ala/Ala genotype. Among control group, 16 (47%) items had Val/Val genotype, 17 (50%) had Val/Ala genotype and 1 (0.02%) had Ala/Ala genotype.

The genotype frequency was different between case and control groups (p=0.0001, Chi-square test). Considering the significance of P-value, the Odd Ratio (OR) CI was calculated by the Madcalc software. The results are shown in table I (OR=0.0168, 95% CI=0.0018-0.153, p=0.0003). The frequency of T and C-alleles was then calculated in both groups. The respective frequency of T and C alleles in the case group was 29% and 71% and it was 73% and 27% in control group (Table I).

**Table I:** The frequency of alleles and genotypes of MnSOD

		**Control group (n=34)**	**Case group (n=34)**	**p-value** *
Genotype		-	-	0.0003
	CC	21 (61)	1 (2)	
	CT	6 (17)	17 (50)	OR = 0.016
	TT	7 (22)	16 (48)	
Allele		-	-	0.0002
	C	24 (71)	9 (27)	-
	T	10 (29)	25 (73)	

Data presented as n (%)

* Statistical analysis was performed using ‎Madcalc (version 12.1) software‎ and Chi squre test.

## Discussion

Based on the results of present study, the Ala/Ala genotype of MnSOD was 61% (21 out of 34 cases). The most important function of MnSOD is its role in the removal of peroxide free radicals to produce H_2_O_2_ and O_2_. The produced H_2_O_2_ will then be decomposed to water by GPX1 and catalase. Therefore, a high percent of MnSOD (in Ala form) may disarrange such balance. Accordingly, H_2_O_2_ will be continually produced but, there are not enough GPX1 and catalase to remove it (22, 23). The result is an imbalance between these three enzymes and concentration of H_2_O_2_ in the cell which endangers DNA (21). It is worth remembering that concentration of H_2_O_2_ in the cells is highly related to many dangerous disorders including an increase of tumor necrosis and apoptosis in cells and may increase cell proliferation rate by creating a protein kinase pathway (21, 22). 

The importance of MnSOD has resulted in investigation of its role in different diseases including breast cancer, prostate cancer, gastric cancer, rectal and colorectal cancer, lung cancer, kidney cancer, many neurodegenerative diseases including Alzheimer and Parkinson, aging process, infertility and spontaneous abortion and also in studying mental and behavioral disorders (12, 16, 22). The results of present study showed the importance of Val16Ala polymorphism of MnSOD in blighted ovum disease. In a study on women with a blighted ovum, it has been shown that echo sound during sonography in patients is different from women with normal pregnancy (20). 

The sounds are weaker in the case of blighted ovum due to the existence of empty pregnancy sac with an approximate size of 2 cm (20). The size of pregnancy sac is one of the most important factors which lead physicians in diagnosing probable disease in pregnant women. A pregnancy sac in women with blighted ovum is about 1.8 cm while in normal cases, it is 1.3 cm. Besides, the surface anatomy of the sac may differ slightly in blighted ovum patients compared to normal pregnancy. Using flexible hysteroscopy, the surface anatomy of the gestational sac and endometrium of blighted ovum and viable pregnancies has been compared. It has been reported that, in general, in blighted ovum cases the sac has lost its surface tension with various degrees, collapsed in shape and its size is smaller compared to the sac in viable pregnancies. In addition, a dark blue color at the sac dome could be observed that is not seen for the sac in viable pregnancies (24).

According to literature, some types of chromosomal abnormalities are the cause of about 40-50% miscarriages (25). However, a more recent review has concluded that both chromosomal and submicroscopic genetic abnormalities are prevalent in about half of the miscarriage samples (26). In order to determine the frequency of balanced chromosomal translocations in diagnosed blighted ovum cases, a study has been reported that among 68 cases 83.4% had normal karyotypes (7). On the other hand, balanced chromosomal rearrangements were relatively low (only 2.3%). It was suggested that single gene determinants may play an important role in such pregnancy complications rather than chromosomal disorders. In contrast to these results, during 2003 a study was performed on 1500 women with blighted ovum who had to undergo an abortion. 61% of them had abnormal karyotype with most common disorders as follows: 52% autosomal trisomy, 20% triploid and 28% monosomy X (5). It has been reported that in many cases of blighted ova both paternal and maternal DNA may have various contributions. Besides, a high incidence of chromosomal abnormalities is also observed in some cases (27). To determine the frequency and type of chromosomal aberrations in different gestational age spontaneous abortions, 106 spontaneous abortions have been studied by comparative genomic hybridization; the highest frequency of chromosome aberrations was observed in blighted ovum specimens compared to other types of spontaneous abortions (28). 

Considering rare literature on MnSOD polymorphism in blighted ovum cases, further investigations are suggested to clearly explore the role of this important antioxidant enzyme in unwanted abortions diagnosed with blighted ovum when considering the higher risks of oxidative stress during pregnancy. Last, not least, it is worth to notice that, although blighted ovum is common, it does not affect the future fertility of patients. The vast majority of women go on to conceive after a blighted ovum miscarriage with no problems. However, a time range of one to three months after miscarrying is recommended before the next pregnancy for the mother to get physically and emotionally ready. In the cases of two or more consecutive miscarriages, a genetic testing may be recommended. In summary, statistical analyses in studied population revealed that MnSOD polymorphism shall be considered as a risk factor in women with a blighted ovum. However, we suggest further studies with larger and wider population.

## Conclusion

Based on the results obtained from our experiments, it is concluded that a significant relationship exists between Val16Ala polymorphism of MnSOD gene and blighted ovum. However, further investigations are required with more cases to clearly explore the role of this important antioxidant enzyme in abortions due to blighted ovum.

## Note

***This article extracted from M.Sc. thesis. (Asieh Moshtaghi)***
